# H2AX deficiency is associated with erythroid dysplasia and compromised haematopoietic stem cell function

**DOI:** 10.1038/srep19589

**Published:** 2016-01-21

**Authors:** Baobing Zhao, Timothy L. Tan, Yang Mei, Jing Yang, Yiting Yu, Amit Verma, Ying Liang, Juehua Gao, Peng Ji

**Affiliations:** 1 Department of Pathology, Feinberg School of Medicine, Northwestern University, Chicago, IL, USA; 2 Department of Developmental and Molecular Biology, Albert Einstein College of Medicine, Bronx, NY, USA; 3 Department of Toxicology and Cancer Biology, University of Kentucky, Lexington, KY, USA

## Abstract

Myelodysplastic syndromes (MDS) are clonal disorders of haematopoiesis characterised by dysplastic changes of major myeloid cell lines. However, the mechanisms underlying these dysplastic changes are poorly understood. Here, we used a genetically modified mouse model and human patient data to examine the physiological roles of H2AX in haematopoiesis and how the loss of H2AX contributes to dyserythropoiesis in MDS. H2AX knockout mice showed cell-autonomous anaemia and erythroid dysplasia, mimicking dyserythropoiesis in MDS. Also, dyserythropoiesis was increased in MDS patients with the deletion of chromosome 11q23, where H2AX is located. Although loss of H2AX did not affect the early stage of terminal erythropoiesis, enucleation was decreased. H2AX deficiency also led to the loss of quiescence of hematopoietic stem and progenitor cells, which dramatically compromised their bone marrow engraftment. These results reveal important roles of H2AX in late-stage terminal erythropoiesis and hematopoietic stem cell function.

The maintenance of cell homeostasis requires genomic integrity, and its dysregulation can lead to various cancers and pre-cancer syndromes^1^,^2^. Myelodysplastic syndromes (MDS) are clonal disorders of haematopoiesis, characterised by dysplastic changes in one or more myeloid lineages and an increased risk of developing acute myeloid leukaemia. A key feature of MDS is the heterogeneous nature of their pathogeneses^3^. Different subtypes of MDS often involve cytogenetic abnormalities, including the deletion of chromosome 5q or 7q (del(5q) or del(7q)) or an extra copy of chromosome 8 (trisomy 8), or less frequently, abnormalities of chromosomes 21, 17, 20, or 11^4^. Epigenetic changes and somatic mutations also contribute to the development of MDS^5^,^6^,^7^. Some dysplastic changes are more frequently associated with certain cytogenetic abnormalities or genetic mutations. For example, mononuclear megakaryocytes are often observed in MDS patients with del(5q)^8^,^9^,^10^, and mutations in the splicing factor *SF3B1* are frequently associated with ring sideroblasts^3^,^11^,^12^,^13^.

Dysplasia in the erythroid lineage represents a variety of morphological changes in the bone marrow, including nuclear budding or irregular nuclear contour in erythroblasts. In peripheral blood, MDS patients often present with normocytic or macrocytic anaemia with relatively nonspecific findings, such as Howell-Jolly bodies. Howell-Jolly bodies are nuclear remnants in red blood cells that are normally removed by the spleen and are often seen in asplenic patients. However, the presence of Howell-Jolly bodies in MDS is an independent finding, as most patients do not exhibit spleen pathology^14^,^15^,^16^. Although the pathogenesis of Howell-Jolly bodies is unknown, a previous study indicates its association with genotoxic exposure among splenectomised human populations^17^. A more recent study reveals that many Howell-Jolly bodies contain centromeres, most frequently observed in chromosomes 1, 5, 7, 8, and 18^18^. As a fast-dividing cell type in the bone marrow, erythroblasts need to maintain a steady, high rate of differentiation and proliferation to replenish millions of senescent red cells every second. Therefore, a sophisticated regulatory system is required in erythroblasts to maintain genomic integrity and correct errors in the DNA replication.

H2AX is a histone variant with a major function in the DNA double-strand break (DSB) repair^19^,^20^,^21^. Upon DSB induced by internal or external stresses, H2AX is phosphorylated at amino acid 139 on its C-terminal tail and accumulates at the DNA damage site^19^,^22^. Phosphorylated H2AX, also called γ-H2AX, further recruits downstream DNA repair proteins such as 53BP1, MDC1, RAD51, BRCA1, and the MRE11/RAD50/NBS1 complex to DSB sites. Mice with H2AX deficiency show chromosomal instability, DSB repair defects, and impaired recruitment of repair proteins to DSB sites^23^. Furthermore, mice with combined H2AX and TP53 deficiency develop several different cancers, including hematologic malignancies^24^. Together, these studies suggest that loss of H2AX plays a role in the development of myeloid dysplasia. However, the specific function of H2AX in haematopoiesis, and whether the loss of H2AX contributes to MDS pathogenesis, is unknown. Here, using a H2AX knockout mouse model, we discovered key roles of H2AX in maintaining genomic integrity in late-stage terminal erythroblasts and the engraftment of hematopoietic stem cells (HSCs) and progenitors. Data from MDS patients further indicate that loss of H2AX could be a mechanism contributing to dyserythropoiesis in MDS.

## Results

### Loss of H2AX in mice mimics dyserythropoiesis in MDS

**Figure 1:**
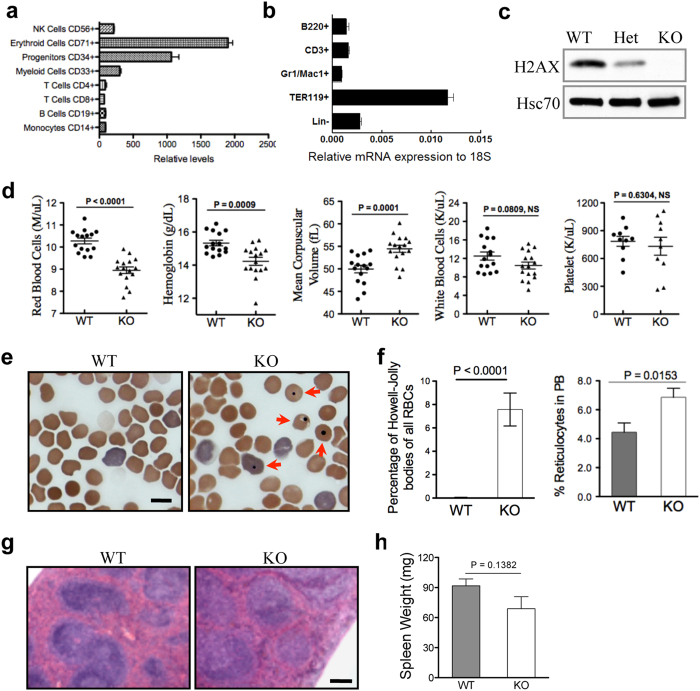
Macrocytic anaemia and increased Howell-Jolly bodies in H2AX-deficient mice. (**a,b**) Relative H2AX mRNA expression in the indicated cells from humans (**a**) and mice (**b**). Data from human mRNA expression were obtained from the Scripps Research Institute BioGPS database^43^. B220, CD3, Gr1/Mac1, Ter119, and Lin- represent B cells, T cells, granulocytes, erythroid cells, and lineage-negative cells, respectively. (**c**) Western blot analysis of H2AX protein expression in mouse bone marrow cells. Hsc70 was used as a loading control. WT = wild-type, Het = heterozygous, KO = knockout. (**d**) Complete blood count. Each dot represents a single mouse (n = 16 per group, except n = 10 per group for platelet count) aged 6 to 8 weeks. (**e**) Representative micrographs of peripheral blood smears from H2AX knockout and wild-type mice. Arrows indicate red blood cells with Howell-Jolly bodies. Scale bar: 10 μm. (**f**) Quantification of red blood cells (RBCs) and peripheral blood (PB) reticulocytes with Howell-Jolly bodies in (**e**) (n = 3 per group). Data are shown as mean ± standard deviation (SD). (**g**) Hematoxylin and eosin staining of spleens from H2AX knockout and wild-type mice. Scale bar: 200 μm. (**h**) Quantification of spleen weight (n = 5 per group). Data are shown as mean ± SD.

To understand the role of H2AX in haematopoiesis, we compared H2AX expression across different blood lineages. Quantitative real-time PCR analysis revealed high H2AX expression in erythroid and hematopoietic progenitor cells in both humans (Fig. 1a) and mice (Fig. 1b). These findings led us to explore the functions of H2AX in erythroid and HSCs/progenitors. We first confirmed H2AX deficiency in the peripheral blood of H2AX knockout mice using western blot analysis (Fig. 1c). Complete blood count showed that H2AX knockout mice exhibited macrocytic anaemia (i.e., increased mean corpuscular volume) but other blood lineages, including white blood cells and platelets, were unaffected (Fig. 1d), consistent with high H2AX expression in the erythroid lineage (Fig. 1b). We next analysed the morphology of peripheral red blood cells and found a significant increase in the percentage of cells with Howell-Jolly bodies and the percentage of reticulocytes in peripheral blood of H2AX knockout mice (Fig. 1e,f). As individuals with surgical removal or severe pathology of the spleen show more Howell-Jolly bodies, we next analysed the size and histology of spleens in H2AX knockout mice. Compared to wild-type mice, there were no pathological or weight changes in spleens from H2AX knockout mice (Fig. 1g,h). These results suggest that H2AX plays a role in normal erythropoiesis.

**Figure 2:**
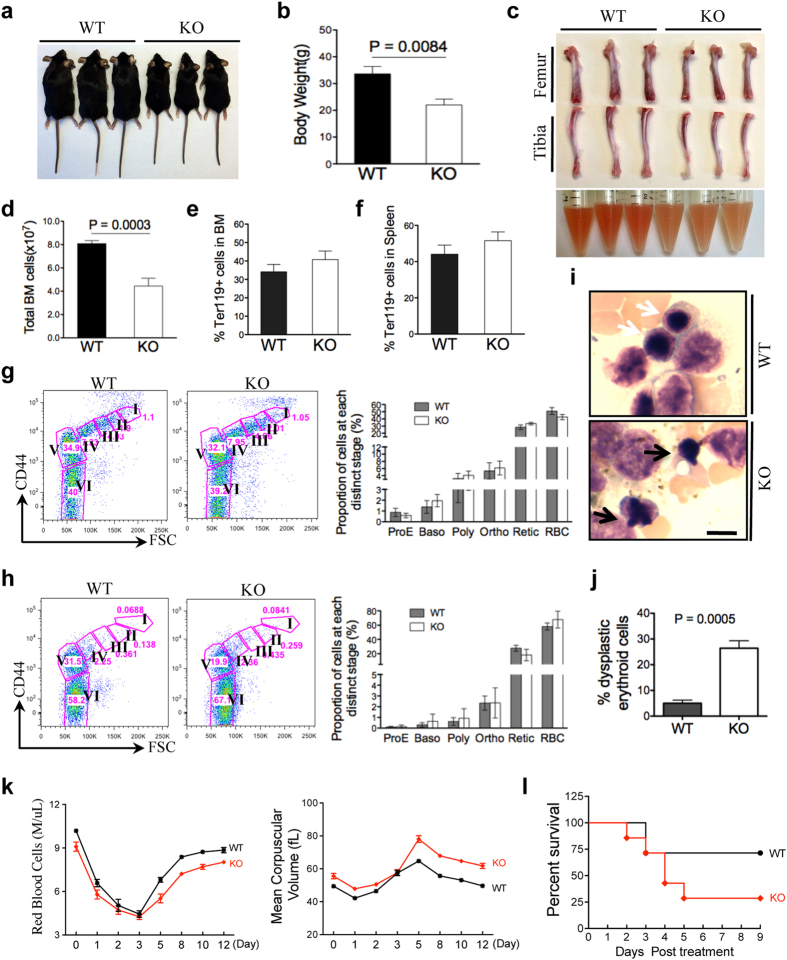
Dysplasia in late-stage terminal erythroblasts in H2AX knockout mice. (**a**) Representative images of H2AX knockout and wild-type mice aged 8 to 12 weeks. (**b**) Quantification of body weight of mice in (**a**). Data are shown as mean ± SD. (**c**) Representative images of femurs and tibias from wild-type and H2AX knockout mice (upper panel). Images of bone marrow cells from two femurs and two tibias per mouse in 1 ml PBS (lower panel; left three tubes: WT, right three tubes: KO). (**d**) Quantification of mononuclear cells in the bone marrow of mice (n = 5 per group). Data are shown as mean ± SD. (**e,f**) Percentage of Ter119-positive cells in the bone marrow (BM; e) and spleen (**f**) was measured by flow cytometry (n = 5 per group). Data are shown as mean ± SD. (**g,h**) Flow cytometric analysis of CD44 and FSC levels of CD45-negative erythroblasts from the bone marrow (**g**) and spleen (**h**) of mice. Populations I–VI represent proerythroblasts, basophilic erythroblasts, polychromatic erythroblasts, orthochromatic erythroblasts, reticulocytes, and red blood cells, respectively. Quantification is shown to the right. (**i**) Wright-Giemsa staining of bone marrow smears from mice aged 8 to 12 weeks. White arrows indicate normal erythroblasts, and black arrows indicate dysplastic erythroblasts with nuclear budding. Scale bar: 10 μm. (**j**) Quantification of dysplastic erythroblasts in (**i**). Data are from 30 high-power bone marrow smear images (n = 3 per group) and are shown as mean ± SD. (**k**) H2AX knockout and wild-type mice were treated with PHZ (50 mg/kg at day 0), and peripheral blood (20 μl per mouse) was collected at the indicated time points. Red blood cells and mean corpuscular volume were measured using an automated hemovet (n = 9 per group). Data are shown as mean ± SD. (**l**) H2AX knockout and wild-type mice were treated with PHZ (100 mg/kg at day 0 and 50 mg/kg at day 1). Survival of recipients (n = 7 per group).

To investigate the pathogenesis of macrocytic anaemia and increased Howell-Jolly bodies associated with the loss of H2AX, we performed comprehensive bone marrow studies comparing wild-type and H2AX knockout mice. As previously reported, H2AX knockout mice were smaller than their wild-type littermates (Fig. 2a,b)^21^,^23^. Accordingly, their bone size and total number of bone marrow cells were also reduced (Fig. 2c,d). However, decreased bone marrow cellularity did not affect the lineage compositions of various hematopoietic cells, including erythrocytes (Fig. 2e and Supplementary Fig. 1). Similarly, the percentage of erythroid cells in the spleen did not differ between wild-type and H2AX knockout mice (Fig. 2f). We further analysed terminal erythropoiesis in bone marrow and the spleen using CD44 and forward scatter (FSC) by flow cytometry^25^. During terminal erythropoiesis, CD44 and FSC gradually decreased in maturing erythroblasts^25^,^26^. Consistent with undisturbed erythroid composition, terminal erythropoiesis was intact in both bone marrow and the spleen in H2AX knockout mice (Fig. 2g,h). These results indicate that loss of H2AX does not affect erythropoiesis *in vivo* under a steady state.

Because Howell-Jolly bodies are frequently observed in MDS patients, we next examined the morphology of bone marrow erythroid cells in H2AX knockout mice. Compared with the round and smooth nuclei of erythroblasts in wild-type mice, erythroblasts in H2AX knockout mice showed frequent nuclear budding with irregular nuclear contours (Fig. 2I,j). Notably, dyserythropoiesis was mostly found in the polychromatic and orthochromatic stages (i.e., late in differentiation) of terminal erythropoiesis, consistent with findings in MDS patients indicating that most dysplastic changes occur in late-stage erythroblasts^15^,^16^. No dysplasia was observed in other myeloid and megakaryocytic lineages (data not shown). Additionally, we found increased 53BP1 focal aggregation in H2AX knockout erythroid cells, indicating possible activation of the DNA damage pathway (Supplementary Fig. 2). Taken together, these results demonstrate that the loss of H2AX in mice mimics the anaemia, dyserythropoiesis, and increased Howell-Jolly bodies observed in MDS patients.

To determine whether loss of H2AX affects erythropoiesis under stress conditions, we challenged mice with two different doses of phenylhydrazine (PHZ). With a low dose, H2AX knockout mice exhibited a poorer rate of recovery of red blood cell indices compared with wild-type mice (Fig. 2k). This compromised response was more prominent when mice were treated with a higher, lethal dose of PHZ, after which H2AX knockout mice showed significantly shortened survival (Fig. 2l). These results indicate that in addition to its role in late-stage terminal erythropoiesis, H2AX is also involved in the response of erythropoiesis to stress.

### Loss of H2AX affects enucleation in late-stage terminal erythropoiesis

**Figure 3:**
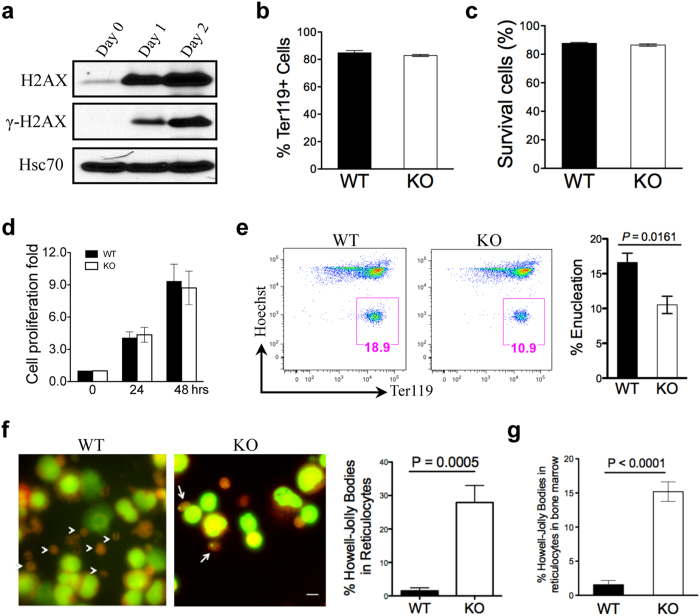
Loss of H2AX affects enucleation in late-stage terminal erythropoiesis. (**a**) Western blot analysis of H2AX and γ-H2AX levels in cultured mouse foetal liver erythroblasts with erythropoietin. Hsc70 was used as a loading control. (**b,c**) After 2 days in culture, cells were harvested. (**b**) Cell differentiation was assessed using flow cytometry to quantify the percentage of Ter119-positive cells. (**c**) Cell survival was assessed quantifying the percentage of annexin V- and propidium iodide-negative cells. (**d**) Quantification of cell growth over time (relative fold change in number of cells). Data are shown as mean ± SD from three independent experiments. (**e**) Flow cytometric analysis of enucleation using Hoechst 33342 and Ter119. The boxed regions with percentages represent enucleated cells. Quantification of enucleated cells is to the right. Data are shown as mean ± SD from three independent experiments. (**f**) Acridine orange staining of cytospin slides of cultured foetal mouse liver cells. Arrowheads indicate reticulocytes, and arrows indicate reticulocytes with Howell-Jolly bodies. Quantification of reticulocytes with Howell-Jolly bodies is to the right. Scale bar: 5 μm. Data were obtained from three independent experiments and are shown as mean ± SD. (**g**) Quantification of reticulocytes with Howell-Jolly bodies in bone marrow smears. Data were obtained from three independent experiments and are shown as mean ± SD.

As a fast-dividing cell type, erythroid cells require tight regulation of genomic integrity^27^. Given the role of H2AX in DNA DSB repair and dyserythropoiesis, H2AX could function in late-stage terminal erythropoiesis, but this might not be adequately detected *in vivo* due to potential compensatory pathways. Therefore, we purified foetal liver erythroblasts and analysed erythropoiesis *in vitro* using a well-established culture system^28^,^29^,^30^. In this model, mouse foetal liver Ter119-negative (an erythroid cell marker) erythroblasts are purified on embryonic day 13.5 and cultured in erythropoietin containing medium for 2 days, after which they mature into Ter119-positive cells with more than 30% of cells enucleated. We first analysed H2AX expression at different stages of terminal erythropoiesis. Levels of total H2AX protein and γ-H2AX were dramatically increased during terminal erythropoiesis (Fig. 3a), consistent with its role in late-stage erythropoiesis. After 2 days, cell differentiation (Fig. 3b), apoptosis (Fig. 3c), and proliferation (Fig. 3d) were undisturbed, but enucleation was significantly reduced in H2AX knockout foetal liver cells (Fig. 3e). Consistent with our *in vivo* finding, the percentage of reticulocytes with Howell-Jolly bodies was significantly increased in H2AX knockout mice (Fig. 3f). As these cells were more numerous than those in the peripheral blood of H2AX knockout mice, many of these abnormal circulating red blood cells may have been removed by the spleen. Indeed, we found more reticulocytes with Howell-Jolly bodies in bone marrow smears from H2AX knockout mice than in peripheral blood (Fig. 3g). Together, these results further confirm that H2AX mainly functions in late-stage terminal erythropoiesis.

### The role of H2AX in late-stage terminal erythropoiesis is cell-autonomous

**Figure 4:**
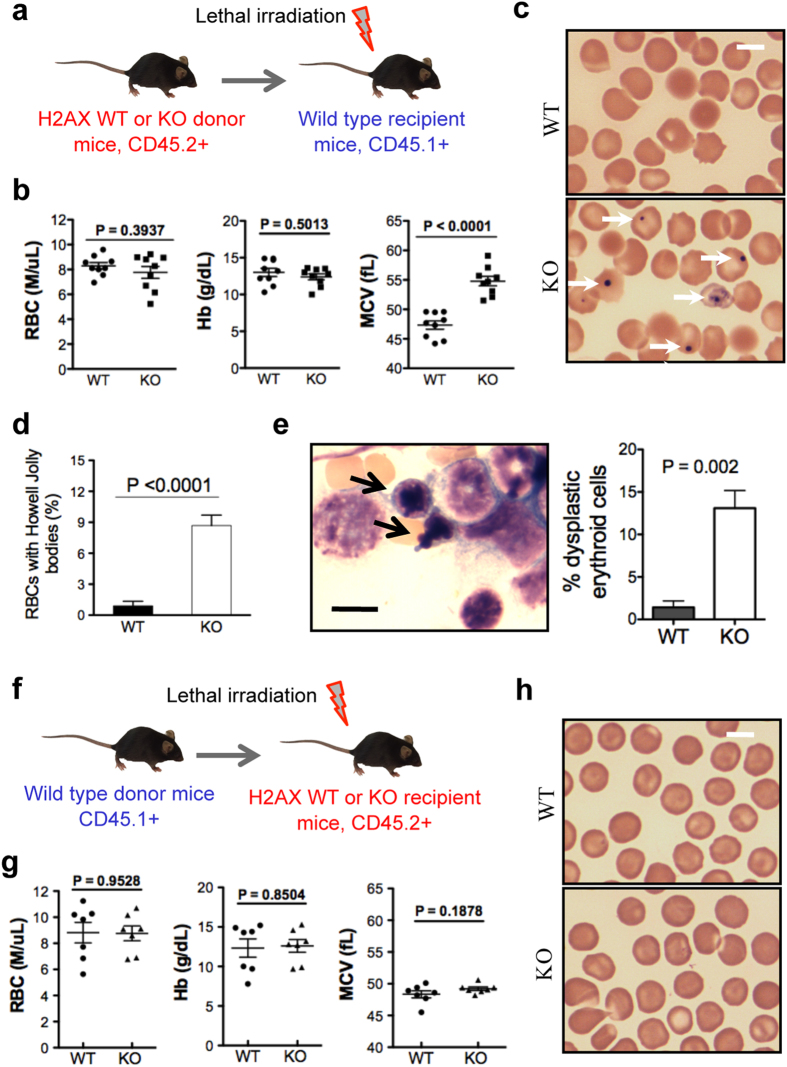
Dyserythropoiesis resulting from loss of H2AX is cell-autonomous. (**a**) Schematic illustration of bone marrow transplantation strategy. (**b**) Peripheral RBC count, haemoglobin (Hb), and mean corpuscular volume (MCV) in recipient mice (CD45.1-positive) 1 month after transplantation of bone marrow cells (CD45.2-negative; n = 9 per group). (**c**) Representative images of peripheral blood smear from mice in (**b**). Arrows indicate red blood cells with Howell-Jolly bodies. Scale bar: 5 μm. (**d**) Quantification of red blood cells with Howell-Jolly bodies in (**c**). Data are shown as mean ± SD. (**e**) Representative image of Wright-Giemsa staining of bone marrow smears of mice transplanted with H2AX knockout bone marrow cells. Arrows indicate dysplastic erythroblasts with nuclear budding. Scale bar: 10 μm. Quantification of the percentage of dysplastic erythroblasts among all bone marrow erythroblasts is shown to the right (n = 3 per group). Data are shown as mean ± SD. (**f**) Schematic illustration of reverse bone marrow transplantation strategy. (**g**) Peripheral RBC count, Hb, and MCV of H2AX knockout and wild-type recipient mice 1 month after transplantation of bone marrow cells (CD45.1-positive; n = 7 per group). (**h**) Representative images of peripheral blood smear from mice in (**g**). Scale bar: 5 μm.

Given the potential effects of the microenvironment on the phenotype of H2AX knockout mice, we next investigated whether dyserythropoiesis induced by the loss of H2AX is cell-autonomous. We performed a transplantation assay in which CD45.2-positive bone marrow cells from H2AX knockout or wild-type mice were transplanted into lethally irradiated CD45.1-positive recipient mice (Fig. 4a). Two months after transplantation, complete blood count was measured together with morphological examination of peripheral blood from recipient mice. Although red blood cell count and haemoglobin level were unaffected, mean corpuscular volume, which reflects the volume of circulating red blood cells, as well as the number of Howell-Jolly bodies were significantly increased in mice transplanted with H2AX-deficient bone marrow cells (Fig. 4b–d). In addition, late-stage erythroblasts in mice transplanted with H2AX-deficient bone marrow cells exhibited dysplastic changes that were similar to those observed in H2AX-null mice (Fig. 4e). These changes in red blood cell counts and erythroblasts were not observed when CD45.1-positive wild-type bone marrow cells were transplanted into lethally irradiated CD45.2-positive H2AX knockout recipient mice (Fig. 4f–h). These results demonstrate that dysplastic changes in the erythroid population of H2AX knockout mice are cell-intrinsic. However, the bone marrow environment could also play a role in the development of anaemia in these mice.

### H2AX deficiency impairs HSC and progenitor cell function

**Figure 5:**
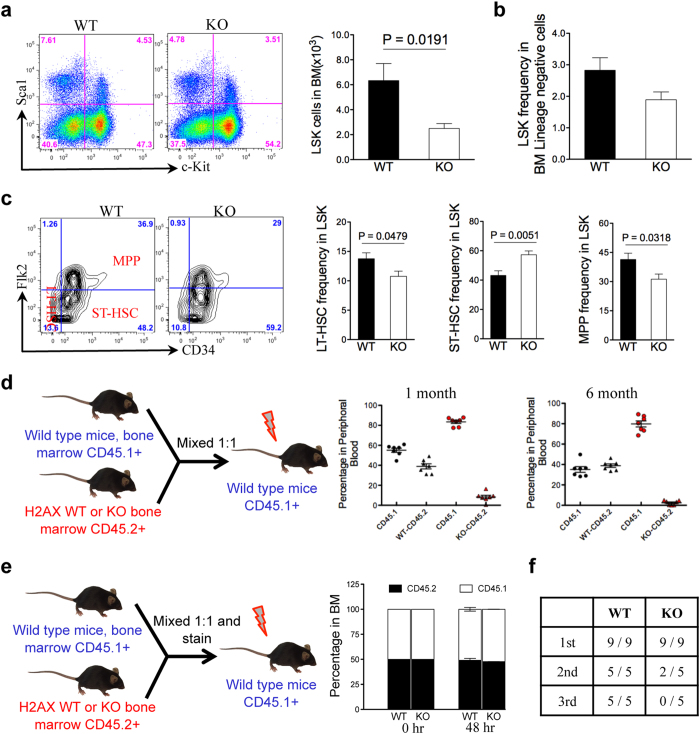
H2AX deficiency impairs HSC/progenitor function. (**a**) Quantification of HSCs/progenitors. Lineage-negative bone marrow cells from 3-month-old H2AX knockout and wild-type mice were isolated and analysed by flow cytometry for the indicated surface markers (n = 5 per group). Quantification of the number of LSK cells is shown to the right. (**b**) Quantification of the percentage of LSK cells (n = 5 per group). (**c**) Representative flow cytometric profiles of long-term HSCs (LT-HSCs), short-term HSCs (ST-HSCs), and multipotent progenitors (MPPs) from pre-gated LSK cells in (**a**). Quantification of the indicated populations is shown to the right. Data are shown as mean ± SD from three independent experiments. (**d**) Schematic illustration of the competitive transplantation assay. Bone marrow from 3-month-old H2AX knockout and wild-type mice (CD45.2-positive) was harvested and mixed with wild-type bone marrow (CD45.1-positive) at a 1:1 ratio and transplanted into lethally irradiated wild-type mice (CD45.1-positive, n = 8 per group). Quantification of donor chimerism in peripheral blood 1 and 6 months post-transplantation is shown to the right. (**e**) Schematic illustration of the homing assay. Bone marrow cells were harvested and mixed as in (**d**). The mixed bone marrow cells were stained *in vitro* and transplanted into lethally irradiated wild type mice (CD45.1-positive, n = 8 per group). Quantification of donor chimerism in bone marrow 48 hours post-transplantation is shown to the right. (**f**) Noncompetitive serial transplants were initiated by transplanting 2 × 10^6^ bone marrow cells from 3-month-old H2AX knockout and wild-type mice (CD45.2-positive) into lethally irradiated recipient mice (CD45.1-positive, n = 8 per group). Secondary and tertiary transplants were performed 4 months later (n = 9 per group in the first transplantation, n = 5 per group in the secondary and tertiary transplantations). Numerator indicates the number of surviving mice.

As H2AX is also highly expressed in HSCs/progenitors, we next tested the function of H2AX in these cells. Consistent with a decreased number of total bone marrow cells, the number of lineage-negative, Sca1- and c-Kit-positive (LSK) cells that comprise relatively enriched HSCs/progenitors was also reduced in H2AX knockout mice (Fig. 5a). However, the percentage of these cells in the bone marrow of H2AX knockout mice was similar to that of wild-type mice (Fig. 5b). We next analysed stem cell populations in the bone marrow and found that the numbers of long-term HSCs and multipotent progenitors were slightly decreased in the H2AX knockout mice, whereas the numbers of short-term HSCs were increased (Fig. 5c). To determine the functional consequences of H2AX deletion on HSCs/progenitors, we performed a competitive transplantation assay, in which equal numbers of wild-type CD45.1-positive bone marrow cells and wild-type or H2AX-knockout CD45.2-positive bone marrow cells were mixed and transplanted into lethally irradiated CD45.1-positive recipient mice (Fig. 5d). Cell engraftment was monitored at different time points by analysing the ratio between CD45.1-positive (wild-type competitor) and CD45.2-positive (H2AX knockout or wild-type testing cells) nucleated cells in peripheral blood. We found that the knockout of H2AX dramatically influenced engraftment 1 and 6 months after transplantation, indicating the impairment of both progenitor and long-term HSC engraftment abilities (Fig. 5d). We confirmed that this compromised engraftment in H2AX-knockout HSCs/progenitors was unrelated to a homing defect (Fig. 5e). The HSC/progenitor cell engraftment defect in H2AX-knockout mice was further validated by a serial transplantation assay, in which H2AX-knockout bone marrow cells rescued lethally irradiated recipient mice in the first transplantation group, but failed to do so in the secondary and tertiary transplantation groups (Fig. 5f). Taken together, these results indicate that H2AX-knockout HSCs/progenitors have severely impaired self-renewal capacity.

**Figure 6:**
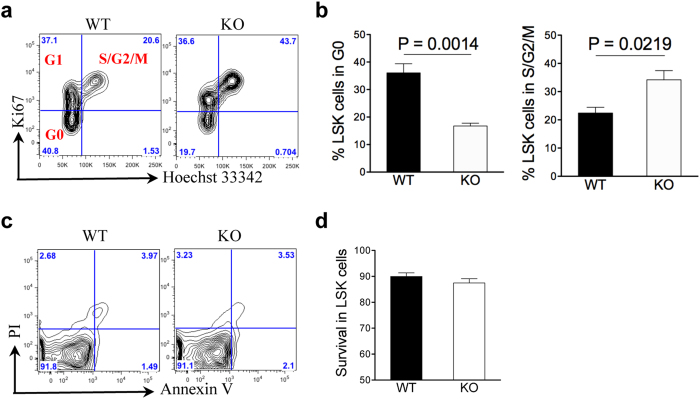
H2AX deficiency induces loss of quiescence in HSCs/progenitors. (**a**) Representative cell cycle profiles of LSK cells from 3-month-old H2AX knockout and wild-type mice analysed by flow cytometry using Hoechst 33342 and Ki67 (n = 4 per group). The percentage of cells in different cell cycle stages is presented. (**b**) Quantification of cells in G0 and G2/S/M phases from (**a**). Data are shown as mean ± SD. (**c**) Apoptosis analysis of LSK cells. The percentage of cells within the LSK fraction is presented. (**d**) Quantification of annexin V and propidium iodide double-negative LSK cells (n = 4 per group). Data are shown as mean ± SD.

Loss of quiescence is a major mechanism of impaired HSC self-renewal, which is observed in mice with targeted deletion of certain DNA repair genes, such as Ku70^31^. To determine whether H2AX deficiency also induces loss of quiescence in HSCs, we analysed the cell cycle profiles of LSK cells in H2AX wild-type and knockout mice. As expected, H2AX knockout mice had more than 2-fold fewer cells in the G0 phase but an increased number of cells in the S/G2/M phase compared with wild-type mice (Fig. 6a,b). Furthermore, apoptosis was comparable between wild-type and knockout LSK cells (Fig. 6c,d). These results demonstrate that knockout of H2AX induces loss of quiescence of HSCs/progenitors, which leads to impaired self-renewal capacities.

### MDS patients with loss of H2AX show increased dyserythropoiesis and poor prognosis

**Figure 7:**
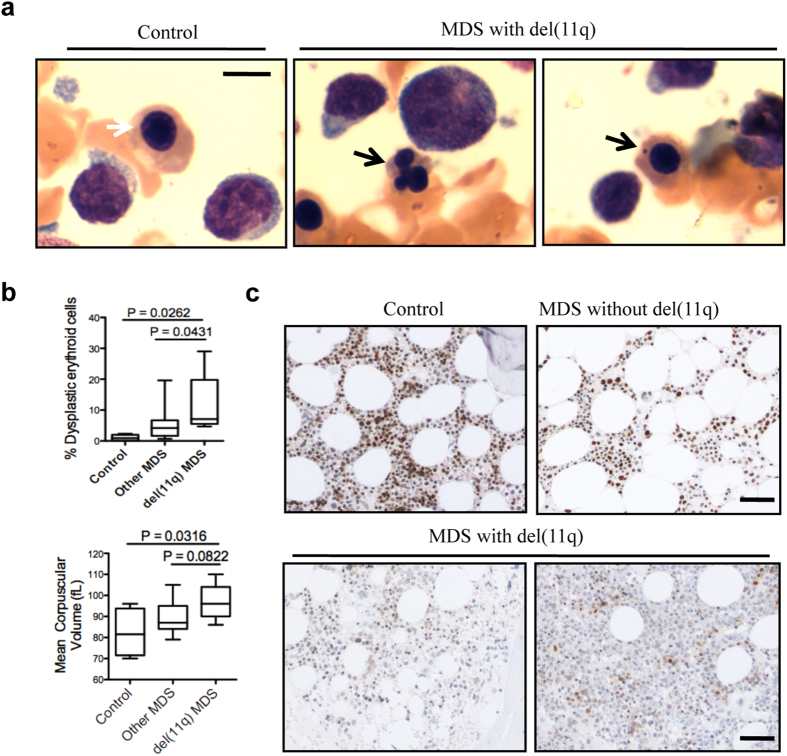
MDS patients with H2AX deficiency exhibit increased dyserythropoiesis and poor prognosis. (**a**) Wright-Giemsa staining of representative bone marrow smears from patients. White arrow indicates a normal orthochromatic erythroblast, and black arrows indicate a dysplastic orthochromatic erythroblast (middle) and an erythroblast with a Howell-Jolly body (right). Scale bar: 10 μm. (**b**) Quantification of dysplastic erythroblasts. Control = bone marrow smears from lymphoma-stage patients, n = 5; Other MDS = bone marrow smears from MDS patients without del(11q), n = 17; del(11q) MDS = bone marrow smears from MDS patients with del(11q), n = 7. (**c**) Immunohistochemical staining for H2AX in bone marrow core biopsies from patients. Scale bar: 100 μm. Control: representative of three lymphoma-stage patients; MDS without del(11q): representative of eight patients; MDS with del(11q): representative of all seven patients. Immunohistochemical staining was performed for all patients, and staining representative of patients with similar results is presented.

In humans, H2AX is located on chromosome 11q23, which is deleted in a small subset of MDS patients with a reported frequency of 0.6%^32^. To determine whether del(11q) is associated with increased dysplasia of erythroid cells, we collocated seven del(11q) MDS cases (both sole del(11q) and in combination with other cytogenetic abnormalities) in the past 5 years from a single medical centre. We then compared the percentage of dysplastic erythroblasts in bone marrow smears from MDS patients with del(11q) to those from MDS patients without del(11q) and lymphoma staging bone marrow patients (i.e., no lymphoma in the bone marrow). As expected, MDS patients with del(11q) had a significantly higher percentage of dysplastic erythroid cells than patients with other MDS forms (Fig. 7a,b). Mean corpuscular volume in patients with del(11q) was significantly higher than that in control patients, and had an increased tendency compared to patients with other MDS forms (Fig. 7b), which is consistent with our findings in H2AX knockout mice. Additionally, there were an increased number of Howell-Jolly body-like micronuclei in many orthochromatic erythroblasts in MDS patients with del(11q) (Fig. 7a). Using immunohistochemical staining, we confirmed that patients with dysplastic erythroid cells and del(11q) exhibited decreased H2AX expression (Fig. 7c).

## Discussion

In this study, we discovered key roles of H2AX in maintaining the genomic integrity of erythroid cells and HSC/progenitor cell function. Loss of H2AX induced dyserythropoiesis mainly in late-stage terminal erythroblasts, consistent with the observation that dysplastic erythroid cells are mostly in polychromatic and orthochromatic stages in MDS patients. Tri-lineage myeloid (granulocytic, erythroid, and megakaryocytic) dysplasia is the hallmark feature and diagnostic criterion of MDS. For the erythroid lineage, dysplastic features include nuclear budding and irregular nuclear contour in bone marrow. We found that these features were recapitulated in late-stage erythroblasts of H2AX knockout mice. We also found increased Howell-Jolly bodies in the red blood cells of H2AX knockout mice. The presence of Howell-Jolly bodies is a nonspecific dysplastic feature of MDS that is not currently listed as a diagnostic criterion. Interestingly, we observed no increase of Howell-Jolly bodies in the red blood cells of MDS patients, but we observed many orthochromatic erythroblasts containing micronuclei resembling Howell-Jolly bodies. These micronuclei were especially prominent in MDS patients with del(11q) and were not observed in normal bone marrow. These findings suggest that the presence of micronuclei in late-stage erythroblasts could be a dysplastic feature of MDS.

In addition to H2AX, other DNA DSB repair proteins are also involved in HSC function^33^,^34^. For example, hypomorphic mutation of DNA ligase IV, a non-homologous end-joining DNA DSB repair protein, causes progressive loss of HSCs and impaired stem cell function^35^. Similarly, loss of Ku70, a component of the non-homologous end-joining DNA repair pathway, induces HSC loss of quiescence, defective self-renewal, and competitive repopulation^31^. Furthermore, the upstream regulator of H2AX, ATM, which phosphorylates H2AX upon DNA damage, also plays an essential role in HSC self-renewal through the regulation of oxidative stress^36^. The defective HSC functions observed in H2AX knockout mice are consistent with these previous findings, thus confirming the critical role of the DNA damage-repair pathway in HSC self-renewal. The prominent dyserythropoiesis resulting from H2AX knockout is unique among these DNA repair proteins, although more careful examination of terminal erythropoiesis needs to be performed in other models.

Deletion of chromosome 11q is infrequent in MDS^32^,^37^. Among multiple genes located on 11q, MLL is particularly important due to its translocation-mediated fusion with many partner genes and its association with acute myeloid leukaemia. To avoid a confounding factor, we excluded patients with MLL translocation from our study. We also confirmed H2AX downregulation in patients with del(11q) using immunohistochemical staining. However, H2AX deletion cannot explain all dysplastic erythroid phenotypes, as other genes located on 11q, such as ATM, could also play a role. Conversely, MDS patients without del(11q) could also have H2AX deletion.

MDS is an age-related clonal HSC disease with a median age of diagnosis of 71 years^16^. Consistently, age-associated functional decline in HSCs is increasingly believed to be involved in the pathogenesis of many myeloid diseases, including MDS^34^. Among many pathophysiological changes during aging, the accumulation of DNA damage is connected with HSC dysfunction, possibly through the loss or mutations of proteins critical for genomic integrity^38^. Aging is also closely associated with diminished erythropoiesis^39^. Although many of these changes are secondary to conditions such as poor nutrition, chronic inflammation, altered humoral homeostasis, and renal insufficiency, cell-intrinsic mechanisms play equally important roles^40^. Similar to HSCs, erythroid progenitor cells accumulate DNA damage during aging. Compared with what is known about DNA damage-repair proteins in HSCs, knowledge of the roles of these proteins in erythropoiesis is scarce. Nevertheless, recent genetic studies show that the loss of PARP-2, a family member of PARP proteins involved in the DNA damage response, causes erythroid dysfunction in mice^40^. In our study, the loss of H2AX affected the function of both HSCs and erythroid progenitors, consistent with age-related deterioration of the DNA damage repair pathway in MDS pathogenesis. For erythropoiesis, H2AX is particularly important for the end stage of differentiation in the form of dysplastic orthochromatic erythroblasts and compromised enucleation. Orthochromatic erythroblasts are tightly linked to enucleation, as most of these end-stage cells exit the cell cycle and prepare to extrude their nuclei. In this respect, it is possible that irregular nuclear contours in orthochromatic erythroblasts in H2AX knockout mice prevent the proper extrusion of nuclei. The formation of Howell-Jolly bodies could also be secondary to nuclear irregularity or partly due to missegregated chromosomes during the final mitosis, as previously reported^41^. Together, these results indicate that enucleation defects could also play a role in the pathogenesis of MDS.

## Methods

### Mice

H2AX knockout mice were generously provided by Dr. Andre Nussenzweig (National Cancer Institute, USA). C57BL/6 mice used for foetal liver cell isolation (CD45.2 background) were purchased from The Jackson Laboratory. B6-LY-5.2/Cr mice (CD45.1 background) used for bone marrow transplantation were purchased from National Cancer Institute. Assays were performed using age- and sex-matched mice, and experimenters were blind to group identities. All mouse studies were performed in accordance with the National Institutes of Health Guide for the Care and Use of Laboratory Animals and were approved by the Institutional Animal Care and Use Committee at Northwestern University.

### Reagents and antibodies

PHZ was obtained from Sigma. Anti-H2AX (Cat. 2595) and anti-phospho-H2AX (Ser139; Cat. 9718) antibodies were obtained from Cell Signaling (Danvers, MA). Anti-HSC70 antibody (Cat. sc-7298) was obtained from Santa Cruz Biotechnologies (Santa Cruz, CA).

### Purification and culture of foetal liver cells

Purification of mouse foetal liver erythroid progenitors (TER119-negative cells) was performed as described previously^29^,^30^. In brief, foetal liver cells were isolated from C57BL/6 mice on embryonic day 13.5 and mechanically dissociated by pipetting in phosphate-buffered saline (PBS) containing 10% foetal bovine serum (Gemini Bio-Products). Single-cell suspensions were prepared by passing the dissociated cells through 40-μm cell strainers (BD Biosciences). Foetal liver cells were labelled with biotin-conjugated anti-TER119 antibody (1:100; eBioscience) and purified using the EasySep column-free cell isolation system (Stem Cell Technologies) according to the manufacturer’s instructions. Purified TER119-negative cells were cultured in erythropoietin (2 unit/ml, PROCRIT®) containing medium.

### Bone marrow transplantation

Bone marrow cells (2 × 10^6^) were purified from H2AX knockout mice and their wild-type littermates (CD45.2-positive) at approximately 6–8 weeks of age. Cells were transplanted into lethally irradiated (10 Gy) recipient mice (CD45.1-positive, B6-LY-5.2/Cr). Recipient mice were then kept on antibiotic water (1.1 mg/ml neomycin [Sigma] and 2000 U/ml polymyxin B [Sigma]) for 2 weeks, followed by regular water. Bone marrow engraftment was analysed by flow cytometric analysis of CD45.2-positive donor-derived cells in the peripheral blood of recipient mice.

For competitive bone marrow transplantation, bone marrow cells (1 × 10^6^) from individual H2AX knockout or wild-type littermate mice (CD45.2-positive) together with an equal number of cells from competitor wild-type mice (CD45.1-positive) were transplanted into lethally irradiated (10 Gy) recipient mice (CD45.1-positive). The ratio of CD45.2- to CD45.1-positive cells in the peripheral blood was measured by flow cytometry.

### Bone marrow homing assay

Bone marrow homing experiments have been described previously^42^. Briefly, bone marrow cells from H2AX knockout or wild-type littermate mice (CD45.2-positive) and competitor wild-type donor mice (CD45.1-positive) were purified and resuspended in ACK lysis buffer (Invitrogen) for approximately 5 minutes on ice with intermittent mixing. Immediately after incubation, cells were washed with ice-cold PBS, counted, mixed at a 1:1 ratio, and stained with DiOC_18_(3) (Invitrogen). Stained bone marrow cells were transplanted into lethally irradiated recipient mice (CD45.1-positive). Bone marrow was collected after 48 h as described above, and the ratio of CD45.2- to CD45.1-positive cells among the DiOC_18_(3)-positive cells was analysed by flow cytometry.

### PHZ-induced stress erythropoiesis

Age- and sex-matched mice were injected intraperitoneally with PHZ (Sigma). Control mice received equal amounts of sterile PBS. Peripheral blood was collected from the tail vein into EDTA-treated tubes and analysed using a Hemavet 950 (Drew Scientific) at different time points.

### MDS patient data

MDS patient data were obtained after informed consent, under the protocols approved by the Institutional Review Board of Northwestern University. The study was conducted in accordance with the Declaration of Helsinki.

### Statistical analysis

Data are shown as mean ± standard error of the mean except when otherwise indicated. Statistical analysis was performed by Student’s t-tests using GraphPad Prism version 6.0 software. A P-value <0.05 was considered statistically significant.

## Additional Information

**How to cite this article**: Zhao, B. *et al.* H2AX deficiency is associated with erythroid dysplasia and compromised haematopoietic stem cell function. *Sci. Rep.* **6**, 19589; doi: 10.1038/srep19589 (2016).

## Supplementary Material

Supplementary figures
