# Supplementary material for: H2AX deficiency is associated with erythroid dysplasia and compromised haematopoietic stem cell function
Source: Sci Rep. 2016 Jan 21;6:19589. doi: 10.1038/srep19589 (PMC4726203; doi:10.1038/srep19589)
Supplement: Supplementary figures [file srep19589-s1.doc]

**Supplementary Information**

**H2AX deficiency is associated with erythroid dysplasia and compromised hematopoietic stem cell function**

Baobing Zhao^1^, Timothy L. Tan^1^, Yang Mei^1^, Jing Yang^1^, Yiting Yu^2^, Amit Verma^2^, Ying Liang^3^, Juehua Gao^1^, and Peng Ji^1^

^1^Department of Pathology, Feinberg School of Medicine, Northwestern University, Chicago, IL, USA

^2^Department of Developmental and Molecular Biology, Albert Einstein College of Medicine, Bronx, NY, USA

^3^Department of Toxicology and Cancer Biology, University of Kentucky, Lexington, KY, USA

**Supplemental Methods**

**Peripheral blood and bone marrow smear with Wright Giemsa and May Grunwald stain**

Freshly collected 2 µl of mouse tail vein blood were smeared over frosted glass slides (VWR International) and air dried, which were then fixed and stained for 5 minutes at room temperature with a readymade Accustain May-Grunwald stain (Sigma). After incubation, the slides were presoaked in 50 mM phosphate buffer of pH 6.8 for 90 seconds and immediately transferred to Accustain Wright-Giemsa stain solution (Sigma). After 20 minutes of incubation the slides were briefly washed with tap water, dried and mounted for visualization under light microscope. For bone marrow smear stain, a wet brush (5/O) presoaked in hypotonic serum (serum to distilled water equals 2:1) was used. The pointed tip of the brush was placed into the cut femur from mouse and mix to create “slurry”. The suspended marrow cells were painted on glass slides using a steady motion, and air-dried completely before proceeding to further steps as detailed above. Peripheral blood, bone marrow smear, and H&E stain micrographs were obtained using an Olympus OX41 microscopeand Infinity 21RC digital camera and analyzed by Infinity analyze software for Mac.

**Flow cytometric analysis**

Flow cytometric analysis of differentiation and enucleation of cultured mouse fetal erythroblasts was performed as previously described (ref 29 in the main text). Flow cytometry was performed on a BD LSR II Fortessa analyzer (BD Biosciences) and the data were further analyzed using FlowJo software. For the analysis of bone marrow cells, total bone marrow cells were flushed from age-matched littermates hind leg bones with PBS (GIBCO) with 2% FBS. Single-cell suspensions were prepared by passing the dissociated cells through 40 μm cell strainers (BD Biosciences). Cells were stained with antibodies specific for cell surface markers in PBS with 2% FBS for 20 min at room temperature, washed and resuspended in PBS.

For the analysis of peripheral blood, approximately 50 µl of tail vein blood were collected in BD microtainer tubes with EDTA (BD Biosciences). The blood was washed with ice cold PBS and resuspended in red blood cell (RBC) lysis buffer for approximately 5 minutes on ice with intermittent mixing. Immediately after incubation, the intact white blood cells were washed with ice cold PBS twice and passed through a 40 µm cell strainer, which were then labeled with appropriate antibodies as detailed in the main text.

For the analysis of cell cycle status, cells were fixed, permeabilized, and labeled with Ki-67-FITC (BD Biosciences) and Hoechst 33342 (Sigma). For apoptosis studies, cells were stained with Annexin V antibody (Biolegend).

**Supplemental Figures**

Granulocyte


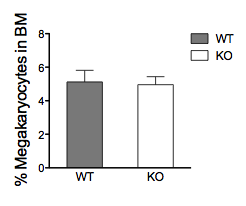


Megakaryocyte


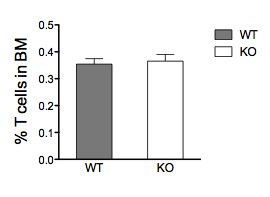


T cells


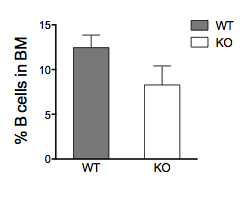


B cells

**Figure S1 H2AX knockout does not affect bone marrow lineage composition.** The percentage of indicated cell lines in the bone marrow was analyzed by flow cytometric assays. N = 5 in each group. Data are presented as mean ± SD.


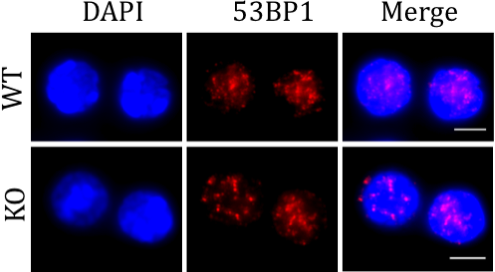


**Figure S2 Immunofluorescence stains for 53BP1 in bone marrow erythroid cells.** Ter119 positive erythroid cells were purified from bone marrow of H2AX knockout mice (KO) and their wild-type littermate controls (WT). The cells were stained as indicated. Scale bars: 5μm.
